# Unified solution of non-limit active and passive earth pressure of retaining wall considering soil arching effect

**DOI:** 10.1371/journal.pone.0326147

**Published:** 2025-06-24

**Authors:** Guihai Gao, Jianxu Chen, Bo Qian, Mei Xiong

**Affiliations:** School of Civil and Hydraulic Engineering, Xichang University, Xichang, Sichuan, China; China University of Mining and Technology, CHINA

## Abstract

The existing theory can not get the unified solution of non-limit active and passive earth pressure with clear mechanical concept. Based on Duncan-Chang stress-strain model, this paper puts forward a relationship between friction angle and wall displacement ratio, which can consider both non-limit active state and non-limit passive state. According to the horizontal layer analysis method, the unified solution of non-limit active and passive earth pressure of retaining wall can be derived, which can be reduced to Rankine solution. The feasibility of the formula is verified by comparing the research results of this paper with the existing theoretical and experimental values. It is found that in the process of changing from non-limit passive state to non-limit active state, the thrust of wall gradually decreases nonlinearly, and the position of the acting point of resultant force gradually increases nonlinearly, and both the change ranges are positively correlated with the internal friction angle. The theoretical formula mechanics in this paper has clear concept and convenient application, which has certain reference significance for engineers.

## 1. Introduction

Accurate calculation of earth pressure is the key to reasonable design of retaining wall. Because classical earth pressure theory [1,2] has a clear mechanical concept and a simple formula, it has always been selected by the majority of engineering practitioners. However, the calculation premise of the classical earth pressure theory is that the backfill behind the wall is in a state of limit equilibrium, and a large number of experiments showed [3–8] that, the magnitude of earth pressure is closely related to displacement and displacement mode of the wall. Therefore, it is of great theoretical and practical significance to research the non-limit earth pressure of retaining walls under different displacement modes. Mei et al. [9] and Xu [10] assumed that the relationship between wall displacement and earth pressure is hyperbolic and trigonometric, respectively, obtaining the formula for calculating earth pressure under arbitrary displacement, but the derivation process is mainly the transformation of mathematical formula, and the mechanical concept is not clear enough. Chang [11] thought that the fundamental reason for the change of earth pressure with displacement was that the internal friction angle of soil gradually developed with the increase of displacement, and assumed the linear relationship between internal friction angle and displacement, and extended Coulomb theory. Chen [12] obtained the hyperbolic relationship between radial stress and strain through unloading triaxial test, establishing the relationship between friction angle and displacement, deduc‌ing the calculation formula of non-limit active earth pressure based on soil wedge balance. Zhang et al. [13] put forward a practical calculation method of seismic earth pressure considering lateral deformation of fill through the coupling effect of compression and shear and the concept of intermediate soil wedge. Wang [14] studied the active earth pressure distribution of retaining wall under translational mode by horizontal layer analysis. The calculated results are basically consistent with the measured values, but the problem of earth pressure coefficient has not been well solved. Chen et al. [15] further explored the influence of the cohesive force of the fill, the corner behind the wall and the inclination angle of the fill surface on the active earth pressure distribution through the upper limit theory and the force balance equation. The numerical solution of the active earth pressure distribution is obtained by Liu et al. [16] considering the influence of the shear stress between the curve slip surface and the soil layer. The results are in good agreement with the experimental results, but iterative calculation is needed. Que et al. [17] studied the formula of active earth pressure thrust in the mode of rotating around the bottom of the wall by slip line theory, but could not get the solution of earth pressure distribution. Lanabi et al. [18] used Flac software to study the passive earth pressure of retaining wall in the modes of rotation around the top of the wall, rotation around the bottom of the wall and translation, and the numerical results obtained were basically consistent with the experimental values. Hu et al. [19] further introduced the influence of displacement on passive earth pressure. With the in-depth study of earth pressure of retaining wall, the influence of soil arching effect on earth pressure has attracted more and more attention of scholars. Handy [20], Paik and Salgado [21] and Goel & Patra [22] assumed that the shape of soil arch is catenary, arc and parabola respectively, and derived the active earth pressure distribution of retaining wall in the limit state. Based on Coulomb slip surface, Cao et al. [23] considered soil arching effect, obtaining the active earth pressure distribution formula that the wall back is inclined by using the curved thin-layer element method. Based on Rankine slip surface, Peng and Zhu [24] considered the shear stress between soil layers, the acquired active earth pressure distribution is basically consistent with the measured value. Based on the curve slip surface, Li et al. [5] got the numerical solution of unlimit active earth pressure by iterative calculation. Based on the plane assumption, Cai et al. [25] derived the formula

that calculates passive earth pressure by using Mohr stress circle. Cao et al. [26] assumed Rankine slip surface and Coulomb slip surface, respectively, setting up the equilibrium equation by using the simplified principal stress trace theory. The two solutions of passive earth pressure distribution proposed are close and basically consistent with the experimental values.

When scholars have studied the non-limit earth pressure, the mechanical concept is not clear enough, moreover, most theories study the (non-limit) active earth pressure and (non-limit) passive earth pressure unilaterally. In fact, the distinction between the non-limit active earth pressure and the non-limit passive earth pressure is that the displacement direction and magnitude are different, and the analysis process is basically similar. Based on this understanding, firstly, through the stress-strain hyperbola model of Duncan-Chang [27], construct the monotonic function relationship between friction angle and displacement ratio by analogy with the change process of wall soil stress with displacement. Then, based on the horizontal layer analysis method and considering the soil arching effect, a unified expression for calculating the active and passive earth pressures under non-limit state is derived. Finally, the experimental values and existing theoretical solutions verify the rationality of the formula in this paper. The formula proposed in this paper is concise and the mechanical concept is clear, which can provide reference for related engineers.

## 2. Relationship between friction angle and wall displacement in non-limit state

**Fig 1:**
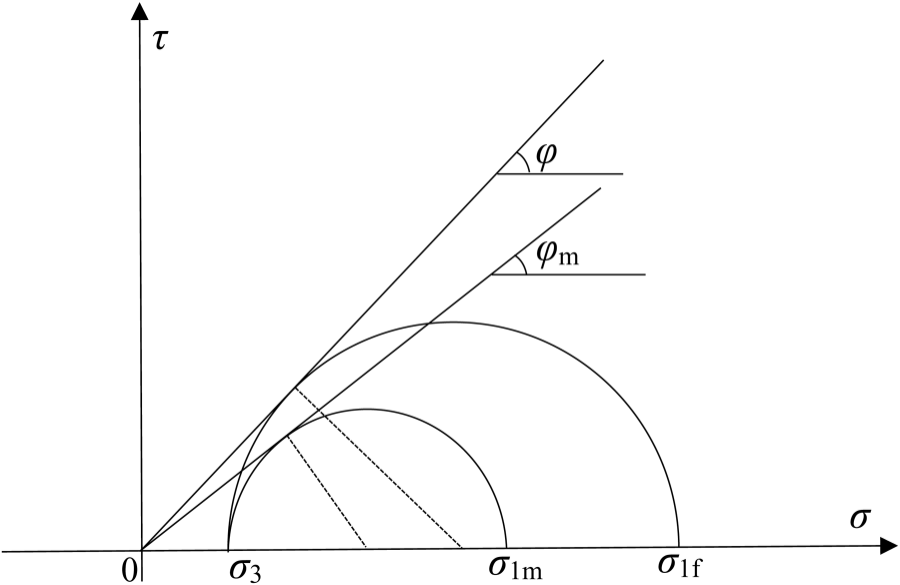
Mohr stress circle under different stress states.


The essential reason why the backfill behind the retaining wall reaches the active limit equilibrium state or the passive limit equilibrium state is caused by the difference between the major and minor principal stresses of the soil. The triaxial shear test process can be analogized to the plastic failure process of the lateral deformation of the soil behind the retaining wall. The research object of this paper is cohesionless soil, and Fig 1 shows the Mohr stress circle of triaxial test of soil. With the increase of principal stress difference, the internal friction angle *φ*_m_ of soil gradually increases from 0 to *φ*. The following relations can be obtained from the diagram.

$$\sin\varphi_{m}=\frac{(\sigma_{1m}-\sigma_{3})/2}{(\sigma_{1m}+\sigma_{3})/2}=\frac{\sigma_{1m}-\sigma_{3}}{\sigma_{1m}+\sigma_{3}}$$ (1)

$$\sin\varphi =\frac{(\sigma_{1f}-\sigma_{3})/2}{(\sigma_{1f}+\sigma_{3})/2}=\frac{\sigma_{1f}-\sigma_{3}}{\sigma_{1f}+\sigma_{3}}$$ (2)

*σ*_1_ represents the major principal stress; *σ*_3_ represents the minor principal stress, *σ*_1m_ represents the major principal stress in the non-limit state; *σ*_1f_ represents the major principal stress in the limit state; *φ* represents the internal friction angle when the soil is destroyed; *φ*_m_ represents the internal friction angle in the non-limit state.

According to Duncan-Chang stress-strain model, the following relationship is satisfied.


$$\sigma_{1m}-\sigma_{3}=\frac{\varepsilon_{m}}{a+b\varepsilon_{m}}$$ (3)

*ε*_m_ represents the axial strain in the non-limit state. *a* and *b* are test parameters related to soil properties. Analysis of formula (3), when *ε*_m_→∞, we can get


$$b=\frac{1}{{\left(\sigma_{1}-\sigma_{3m}\right)}_{\varepsilon_{m}\to \infty }}=\frac{1}{{\left(\sigma_{1}-\sigma_{3m}\right)}_{u}}$$ (4)

(*σ*_1_-*σ*_3m_)_*u*_ is the asymptote value of (*σ*_1_-*σ*_3m_).

The failure ratio R_f_ of soil is introduced, and the expression is


$$R_{f}=\frac{{\left(\sigma_{1m}-\sigma_{3}\right)}_{f}}{{\left(\sigma_{1m}-\sigma_{3}\right)}_{u}}=\frac{\sigma_{1f}-\sigma_{3}}{{\left(\sigma_{1m}-\sigma_{3}\right)}_{u}}$$ (5)

The value of R_f_ is generally 0.75–1.0. When there is no measured value, 0.85 can be taken.

According to formulas (2), (4) and (5), we can get


$$b=\frac{1-\sin\varphi }{2\sigma_{3}\sin\varphi }R_{f}$$ (6)

It can be obtained from formula (1), (2), (3) and (6), namely


$$\varepsilon_{m}=\frac{2a\sigma_{3}\varphi_{m}}{1-\sin\varphi_{m}-R_{f}\frac{1-\sin\varphi }{\sin\varphi }\sin\varphi_{m}}$$ (7)

$$\varepsilon_{f}=\frac{2a\sigma_{3}\varphi_{m}}{\left(1-\sin\varphi )(1-R_{f}\right)}$$ (8)

*ε*_f_ refers to the axial strain in the limit state. Radial strain *ε*_rm_ and axial strain *ε*_m_ satisfy the following relationship,


$$\varepsilon_{rm}=-\mu \varepsilon_{m}$$ (9)

Where *μ* is Poisson’s ratio of soil. Let $\eta =\frac{\varepsilon_{rm}}{\varepsilon_{rf}}=\frac{\varepsilon_{m}}{\varepsilon_{f}}$, from formula (7) and (8), and we can get


$$\eta =\frac{\left(1-\sin\varphi )(1-R_{f}\right)}{1-\sin\varphi_{m}-R_{f}\frac{1-\sin\varphi }{\sin\varphi }\sin\varphi_{m}}$$ (10)

Expression of *φ*_m_ can be obtained from formula (10)


$$\varphi_{m}=\arcsin\left[\frac{\eta \sin\varphi }{1-(1-\eta )R_{f}-(1-\eta )(1-R_{f})\sin\varphi }\right]$$ (11)

For the soil behind the retaining wall, *η* in formula (11) is the displacement ratio, which indicates the ratio of the horizontal displacement value *S* of the wall to the displacement value *S*_u_ in the limit state. For the active state, as the retaining wall moves, the force variation of the backfill behind the wall can be compared by first conducting *K*_0_ consolidation and then performing a triaxial test of the shear stress path that maintains the axial stress unchanged and reduces the radial stress. From the initial state to the failure state, the lateral deformation of the soil sample gradually increases from 0 to the maximum value *S*_u_, which is equivalent to the wall reaching the active limit displacement again from rest. For the same type of soil, when conducting triaxial shear tests with the same initial dimensions, the ratio of the strain *ε*_m_/*ε*_f_ is equal to the ratio of the deformation *S*/*S*_u_, that is, both can be expressed by the wall displacement ratio *η*. Similarly, the difference between the passive state and the active state lies in the different directions of displacement.

If the non-limit active state is specified to be positive, the non-limit passive state is negative. According to classical earth pressure theory [1,2], the wall reaches the active limit equilibrium state and the passive limit equilibrium state respectively, and the active and passive earth pressures can be unified by changing the positive and negative internal friction angles. Given this understanding, if the active limit equilibrium state is considered as *φ*, the passive limit equilibrium state should be considered as -*φ*. Equation (11) can be applied to the non-limit active and passive states at the same time, that is


$$\varphi_{m}=\left\{ \begin{array}{c} \arcsin\left[\frac{\eta \sin\varphi }{1-(1-\eta )R_{f}-(1-\eta )(1-R_{f})\sin\varphi }\right],\eta \in [0,1]\\ \arcsin\left[\frac{\eta \sin\varphi }{1-(1+\eta )R_{f}-(1+\eta )(1-R_{f})\sin\varphi }\right],\eta \in [-1,0) \end{array}\right.$$ (12)

In formula (12), if *η* = 0, that is, the wall does not move at all, then *φ*_m_ = 0, which is inconsistent with the reality. Because if the actual retaining wall is at rest, there will be a principal stress difference, and *φ*_m_ will have a certain initial value *φ*_0_, which can be obtained by the Mohr stress circle in Fig 1. When the wall is in a state of rest, for normally consolidated soil, σ_3_=(1-sinφ) σ_1m_ [28]. Substituting this formula into formula (1), we can obtain


$$\varphi_{0}=\arcsin\left(\frac{\sin\varphi }{2-\sin\varphi }\right)$$ (13)

Considering that *φ*_m_ is a monotonic continuous function of *η*, and *φ* = *φ*_0_ when *η* = 0, Equation (12) can be modified as follows


$$\varphi_{m}=\left\{ \begin{array}{c} \arcsin\left[\frac{\eta \sin(\varphi -\varphi_{0})}{1-(1-\eta )R_{f}-(1-\eta )(1-R_{f})\sin\varphi }\right]+\varphi_{0},\eta \in [0,1]\\ \arcsin\left[\frac{\eta \sin(\varphi +\varphi_{0})}{1-(1+\eta )R_{f}-(1+\eta )(1-R_{f})\sin\varphi }\right]+\varphi_{0},\eta \in [-1,0) \end{array}\right.$$ (14)

**Fig 2:**
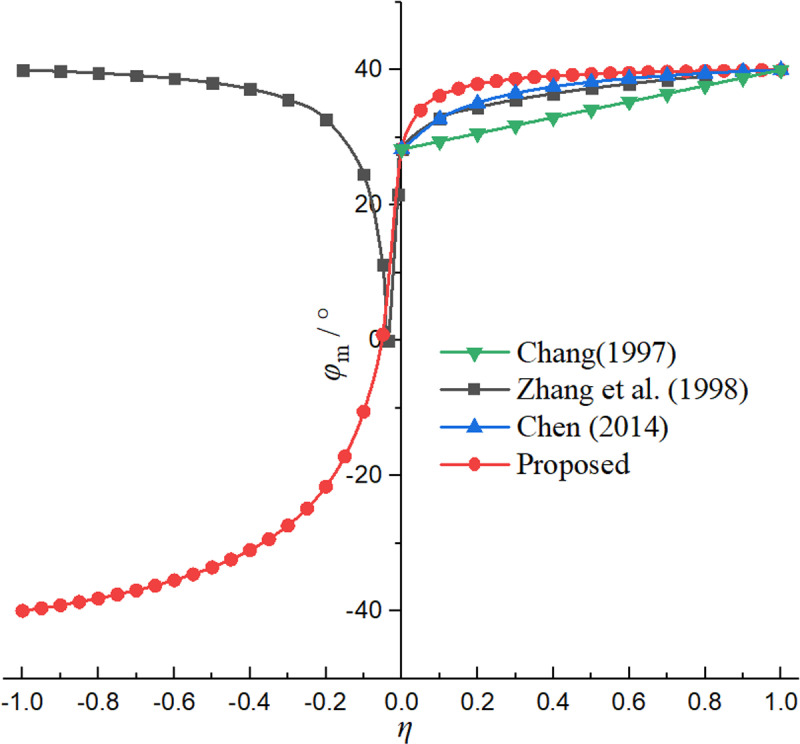
Relationship curves of *φ*_m_ **and** *η* under different theories.

In order to compare the solution in this paper with the existing theoretical solutions, Table 1 lists the *φ*_m_ calculation formulas under three theories: Chang (1997), Zhang et al.(1998), and Chen (2014). Fig 2 shows the relationship between *φ*_m_ and *η* under different theories. It can be seen that Chang [11] and Chen [12] obtained the linear relationship and nonlinear relationship between *φ*_m_ and *η*, respectively, but they can only be applied to the non-limit active state. The relationship curve obtained by Zhang et al. [13] can consider both non-limit active and passive states, but it is impossible to analyze the unified solution of non-limit active and passive earth pressures, and there are more empirical parameters. The curve obtained by the proposed solution is monotonic and continuous, and *φ*_m_ changes from -*φ* to *φ*, which lays a theoretical foundation for the subsequent derivation of the unified solution of non-limit active and passive earth pressures.

**Table 1:** The *φ*_m_ calculation formulas under three theories.

Chang (1997)	$\varphi_{m}=\arctan[\tan\varphi_{0}+\eta (\tan\varphi -\tan\varphi_{0})]$
Zhang et al.(1998)	$\varphi_{m}=\left\{ \begin{array}{c} \arcsin\left[\frac{\sin\varphi (1-R)}{2-\sin\varphi (1+R)}\right]\;\;(-1\leq R\leq 1nonumber\\ \arcsin\left[\frac{\sin\varphi (R-1)}{2-\sin\varphi (3-R)}\right]\;\;\;\;\;(1<R\leq 3) \end{array}\right.$ Where, Non-limit active state: $R=\left\{ \begin{array}{c} -(-\eta {)}^{0.5},-1<\eta \leq 0\\ -1\;\;\;,\eta \leq -1 \end{array}\right.$ Non-limit passive state: $R=\left\{ \begin{array}{c} \frac{\eta }{0.025(1-\eta )+\eta /3}\;,0<\eta <1\\ 3\;\;\;\;\;\;\;,\eta \geq 1 \end{array}\right.$ In the formula, *R* refers the strain constraint parameter.
Chen (2014)	$\sin\varphi_{m}=\frac{2\sin\varphi_{0}(1+\sin\varphi )\left[1-(1-\eta )R_{f}\right]+\eta \sin\varphi (1+\sin\varphi_{0})(1-\sin\varphi )}{2(1+\sin\varphi )\left[1-(1-\eta )R_{f}\right]-\eta \sin\varphi (1+\sin\varphi_{0})(1-\sin\varphi )}$

For the problem of the developed value *δ*_m_ of the wall-soil friction angle, according to the suggestion of Li et al. [5], namely


$$\delta_{m}=\xi \varphi_{m}$$ (15)

where, ξ is the ratio of wall-soil friction angle to internal friction angle, which can be obtained through experiments. If there is no measured value, *ξ* can be taken as 2/3.

## 3. Theoretical derivation of unified solution of earth pressure

When the backfill behind the retaining wall is in an active limit equilibrium state, it can be assumed that the sliding surface behind the wall is plane, which is generally an active Coulomb sliding surface [12,14], active Rankine slip surface [21,24,29]. Hu et al. [15] found the angle between the slip surface of sandy fill and the horizontal plane is about π/4 + φ/2 through experiments. When the backfill behind the retaining wall is in a passive limit equilibrium state, Lanabi [18] found through numerical simulation that the slip surface of sand is slightly curved near the bottom of the wall, and the angle between the upper part and the horizontal plane is π/4-*φ*/2. Cao et al. [26] theoretically deduced that the calculation results of passive Coulomb slip surface and passive Rankine slip surface are close. To sum up, in order to simplify the calculation, Rankine slip surface is applied to the non-limit state in this paper, that is


$$\beta_{m}=\frac{\pi }{4}+\frac{\varphi_{m}}{2}$$ (16)

*β*_m_ is the angle between the slip surface and the horizontal plane in the non-limit state.

**Fig 3:**
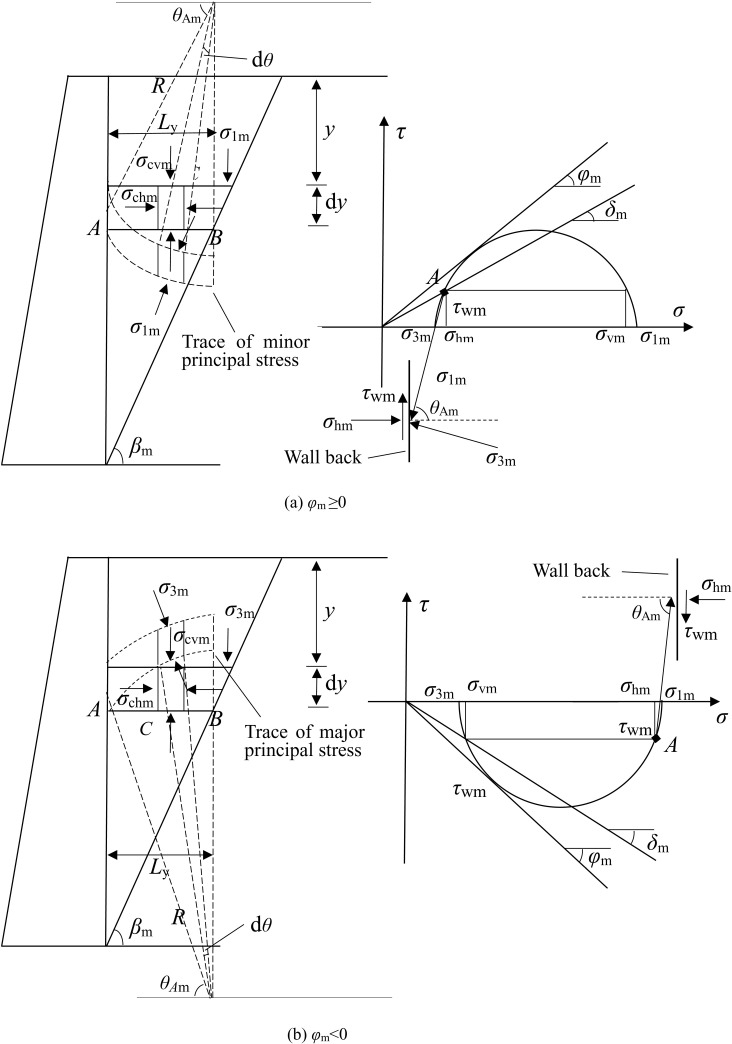
Schematic diagram of calculation model in non-limit state.

The “soil arching effect” is a relatively common phenomenon in geotechnical engineering, where stress redistribution in the soil is caused by stress deflection. In the active state, the backfill behind the wall has a tendency to slide down along the sliding surface. The soil particles move and squeeze each other, causing the minor principal stress (horizontal stress) to gradually form a concave arch. The stress is transferred to the stable soil through this arch structure, as shown in Fig 3a. In the passive state, the backfill behind the wall has a tendency to slide up along the sliding surface. The internal particles of the soil are relatively displaced due to compression, causing the major principal stress (horizontal stress) to gradually form a convex arch, diffusing the wall thrust to the surrounding soil through the arch structure, as shown in Fig 3b.

It is assumed that the soil arch generated in the non-limit state is circular [5,19,21,23,25,26] as shown in Fig 3a, b, has a soil arch radius of *R*. As can be seen from the figure, whether *φ*_m_ ≥ 0 or *φ*_m_ < 0, the length Ly of any differential unit *AB* can be expressed as


$$L_{y}=R\cos\theta_{Am}$$ (17)

*θ*_Am_ is the angle between *OA* and horizontal plane, and its magnitude can be obtained according to the geometric relationship of Mohr stress circle.


$${\theta_{A}}_{m}=\frac{1}{2}\left[\pi -\arcsin\left(\frac{\sin\delta_{m}}{\sin\varphi_{m}}\right)+\delta_{m}\right]$$ (18)

According to Mohr stress circle, horizontal stress at point A can be given.


$$\sigma_{hm}=\left\{ \begin{array}{c} \frac{{\sigma_{1}}_{m}}{1+\sin\varphi_{m}}(1+\sin\varphi_{m}\cos2\theta_{Am}),\varphi_{m}\geq 0\\ \frac{{\sigma_{3}}_{m}}{1+\sin\varphi_{m}}(1+\sin\varphi_{m}\cos2\theta_{Am}),\varphi_{m}<0 \end{array}\right.$$ (19)

The vertical stress at any point *C* on *AB* is


$$\sigma_{cvm}=\left\{ \begin{array}{c} {\sigma_{1}}_{m}\left(1-\sin\varphi_{m}\cos2\theta \right),\varphi_{m}\geq 0\\ {\sigma_{3}}_{m}\left(1-\sin\varphi_{m}\cos2\theta \right),\varphi_{m}<0 \end{array}\right.\;$$ (20)

The average vertical stress can be obtained because the vertical stress at any point *C* on infinitesimal *AB* is different.


$${\bar{\sigma }}_{vm}=\left\{ \begin{array}{c} \frac{\int_{\theta_{Am}}^{\frac{\pi }{2}}\sigma_{cvm}R\sin\theta d\theta }{L_{y}}\;=\;\;\left[1-\frac{2\sin\varphi_{m}\cos^{2}\theta_{Am}}{3(1+\sin\varphi_{m})}\right]{\sigma_{1}}_{m},\varphi_{m}\geq 0\\ \frac{\int_{\theta_{Am}}^{\frac{\pi }{2}}\sigma_{cvm}R\sin\theta d\theta }{L_{y}}\;=\;\;\left[1-\frac{2\sin\varphi_{m}\cos^{2}\theta_{Am}}{3(1+\sin\varphi_{m})}\right]{\sigma_{3}}_{m},\varphi_{m}<0 \end{array}\right.$$ (21)

According to formulas (19) and (21), the lateral earth pressure coefficient *K*_m_ of soil in non-limit state can be given, namely


$$K_{m}=\frac{\sigma_{hm}}{{\bar{\sigma }}_{vm}}=\frac{1+\sin\varphi_{m}\cos2\theta_{Am}}{1+\sin\varphi_{m}-2\sin\varphi_{m}\cos^{2}\theta_{Am}/3}$$ (22)

When Rankine hypothesis is satisfied, the following formula can be obtained according to formulas (14), (18) and (22)


$$K_{m}=\left\{ \begin{array}{c} \frac{1-\sin\varphi }{1+\sin\varphi },\delta =0,\eta =1\\ \frac{1+\sin\varphi }{1-\sin\varphi },\delta =0,\eta =-1 \end{array}\right.$$ (23)

It shows that Rankine earth pressure coefficient is a special case of this paper.

**Fig 4:**
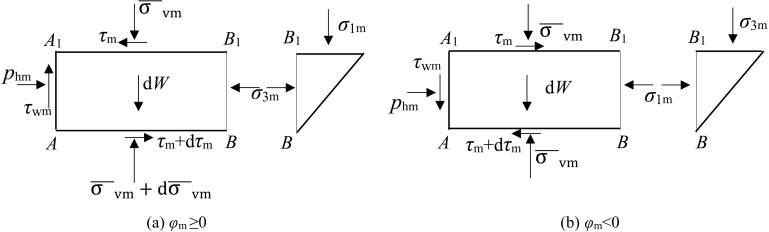
Force analysis of soil differential element.


As shown in Fig 4, the differential element of soil is divided into two parts: rectangle and triangle, and *BB*_1_ is the principal stress plane, so It is not subjected to shear stress. Considering the vertical force balance, because the difference between *φ*_m _≥ 0 and*φ*_m_ < 0 is that the direction of *τ*_wm_ is different, and τ_wm_ = *K*_m_p_hm_tan*δ*_m_, where *δ*_m_ and *φ*_m_ are of the same sign, the vertical force balance equation can be unified as follows

$$K_{m}{\bar{\sigma }}_{vm}\tan\delta_{m}dy+L_{y}d{\bar{\sigma }}_{vm}-dW=0$$ (24)

Where, the soil weight d*W* = *γ*d*y*, *γ* refers to unit weight of soil.

In fact, the expression of ${\bar{\sigma }}_{vm}$ can be derived from differential Equation (24), and the horizontal non-limit earth pressure *p*_hm_ can be given, thus avoiding solving horizontal shear stress τ_m_. Since the current research on the shear force between soil layers is not in-depth, considering only the vertical equilibrium equation can avoid the large calculation error caused by the improper consideration of shear stress to a certain extent.

Solve the differential Equation (24), and combine with the boundary condition, *y* = 0, ${\bar{\sigma }}_{vm}=q$, and we have


$${\bar{\sigma }}_{vm}=\frac{\gamma H}{1-K_{am}\tan\delta_{m}\tan\beta_{am}}\left[{\left(1-\frac{y}{H}\right)}^{K_{m}\tan\delta_{m}\tan\beta_{m}}-(1-\frac{y}{H})\right]+q{\left(1-\frac{y}{H}\right)}^{K_{m}\tan\delta_{m}\tan\beta_{m}}$$ (25)

The horizontal non-limit earth pressure *p*_hm_ is


$$p_{hm}=\frac{\gamma HK_{m}}{1-K_{m}\tan\delta_{m}\tan\beta_{am}}\left[{\left(1-\frac{y}{H}\right)}^{K_{m}\tan\delta_{m}\tan\beta_{m}}-(1-\frac{y}{H})\right]+K_{m}q{\left(1-\frac{y}{H}\right)}^{K_{m}\tan\delta_{m}\tan\beta_{m}}$$ (26)

When Rankine hypothesis is satisfied, formulas (27) can be obtained according to formulas (23) and (26), namely


$$p_{hm}=\left\{ \begin{array}{c} \frac{1-\sin\varphi }{1+\sin\varphi }\gamma z,\delta =0,q=0,\eta =1\\ \frac{1+\sin\varphi }{1-\sin\varphi }\gamma z,\delta =0,q=0,\eta =-1 \end{array}\right.$$ (27)

It shows that Rankine earth pressure is the special solution of this paper.

The resultant force *P*_m_ of non-limit earth pressure is


$$P_{m}=\int_{0}^{H}\frac{\sigma_{hm}}{\cos\delta_{m}}dy=\frac{\gamma H^{2}}{2\cos\delta_{m}}\frac{K_{m}}{1+K_{m}\tan\delta_{m}\tan\beta_{m}}+K_{m}qH$$ (28)

The height *h*_m_ from the resultant force application location to the bottom of the wall in the non-limit state is


$$h_{m}=\frac{\int_{0}^{H}\sigma_{hm}(H-y)dy}{P_{hm}}=\frac{2(1+K_{m}\tan\delta_{m}\tan\beta_{m})}{3(2+K_{m}\tan\delta_{m}\tan\beta_{m})}\left[\frac{\gamma H^{2}+6qH}{\gamma H+6q(1+K_{m}\tan\delta_{m}\tan\beta_{m})}\right]$$ (29)

## 4. Experimental verification

### 4.1. Experiment 1

**Fig 5:**
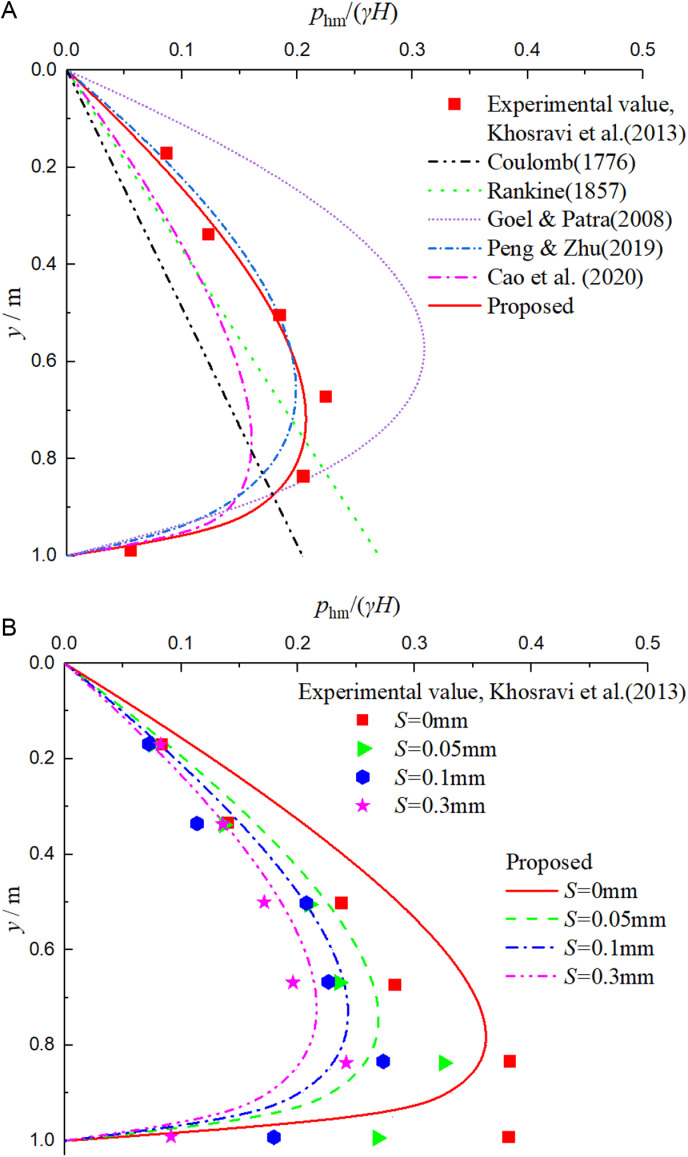
Comparison curves between theoretical solution and experimental value of non-limit active earth pressure under different displacements.

Khosravi et al. [4] explored the distribution rule of non-limit active earth pressure under different displacements through indoor model tests, with test parameters of *H* = 300 mm, *φ* = *δ* = 35°, *ξ*  = 1, *γ* = 12.7kN/m^3^, *q* = 0 and *S*_u_ = 0.525 mm. Fig 5a, b are graphs showing the comparison between the theoretical solution of non-limit active earth pressure and the experimental values under different displacements, and the earth pressure values in the figures are normalized.

As can be seen from Fig 5a, when the fill is in the limit state, the proposed solution and Peng & Zhu solution [24] are in good agreement with the experimental values. In contrast, in the middle and lower parts of the wall, the proposed solution is closer to the measured values, which shows the reliability of this method. The difference between this solution and Peng & Zhu solution [24] is that Peng & Zhu solution [24] does not reasonably consider the shear stress between soil layers, while the proposed solution avoids the solution of horizontal shear stress by balancing the vertical force. Geol & Patra solution [22] is obviously larger than the measured value, mainly because the soil arch is assumed to be parabolic in the derivation process. Cao et al. solution [23] derived the calculation formula for active earth pressure by assuming the Coulomb slip surface and based on the theory of curved thin-layer elements. This led to the obtained earth pressure values being significantly smaller than the experimental values. Coulomb solution [1] and Rankine solution [2] are linearly distributed along the wall height, which is quite different from the measured law.

As can be seen from Fig 5b, when the fill is in the non-limit state, the distribution law of non-limit active earth pressure under different displacements is basically consistent with the measured value, which shows that the relationship between the internal friction angle *φ*_m_ and the displacement ratio *η* deduced in this paper is reasonable. Near the bottom of the wall, the solution in this paper is smaller than the measured value. There are two main reasons. One is that slip surface considered in this paper is planar. Second, the soil near the bottom of the wall is severely constrained in the experiment, which prevents the internal friction angle from being fully exerted. As a result, the developed value of the internal friction angle is relatively small, leading to a larger measured earth pressure value.

### 4.2. Experiment 2

Fang et al. [3] explored the distribution rule of non-limit passive earth pressure under different displacement conditions through indoor model tests, and the test parameters were *H* = 0.5m, *φ* = 33°, *δ* = 9.8°, *ξ*  = 0.297, *γ* = 15.7kN/m^3^, *q* = 0, and *S*_u_ = 0.17*H*. Fig 5a, b are graphs showing the comparison between theoretical solution and experimental value of non-limit passive earth pressure under different displacement conditions.

**Fig 6:**
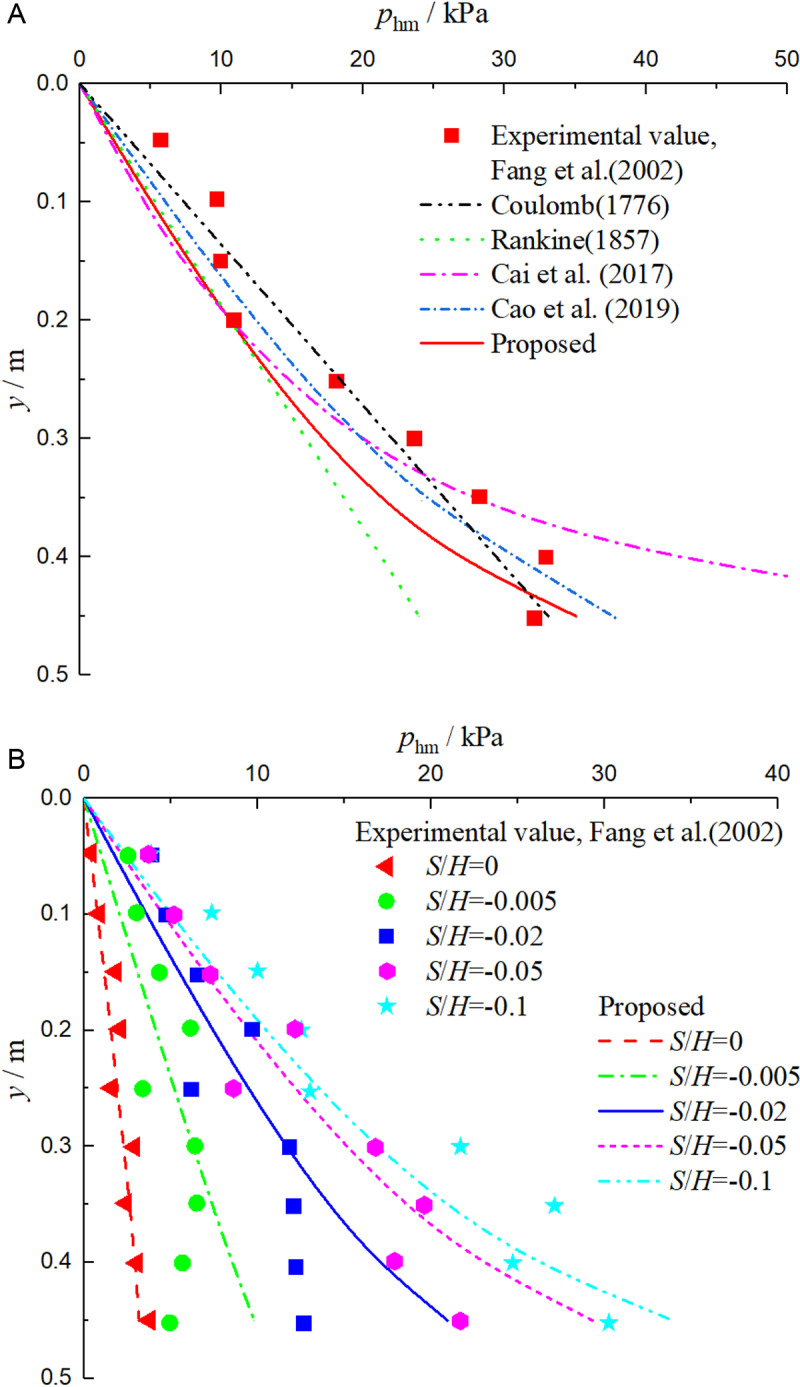
Comparison curves between theoretical solution and experimental value of non-limit passive earth pressure under different displacements.

As can be seen from Fig 6a, when the fill is in the limit state, the measured values are non – linear, while the Coulomb solution [1] and Rankine solution [2] are linear along the wall height, which is inconsistent with the measured trend. The solutions in this paper, Cai et al. solution [25] and Cao et al. solution [26] are all non-linear, which are roughly consistent with the measured values. Cai et al. solution [25] is quite different from the measured value near the wall bottom, mainly due to the unreasonable consideration of the slip surface. The solution in this paper is slightly smaller than that of Cao et al. solution [26], because Cao et al. solution [26] deduced the earth pressure according to the principal stress trace, and the pushing process is extremely complicated, making it impossible to calculate the infinite passive earth pressure. The solution in this paper is generally smaller than the measured value, mainly for two reasons. First, the slip surface is approximately considered as a plane in this paper; Second, the model test is carried out in the three – dimensional state. In the passive state, the wall pushes the soil, resulting in the measured passive earth pressure being obviously larger.

As can be seen from Fig 5b, when the fill is in the non-limit state, the distribution law of non-limit passive earth pressure under different displacements is basically consistent with the measured values, which are located on both sides of the theoretical distribution curve in this paper, and can basically reflect the variation law of earth pressure with displacement, which shows that the relationship between the internal friction angle *φ*_m_ and the displacement ratio *η* deduced in this paper is reasonable.

## 5. *P*_m_ and *h*_m_ under different *S* and *φ*

In order to explore the variation law of the non-limit earth pressure *P*_m_ and the action point *h*_m_ of the resultant earth pressure under different displacements and internal friction angles, it is assumed that the displacements required for the soil to reach the active limit equilibrium state and the passive limit equilibrium state are 0.001*H* and 0.01*H* respectively.

**Fig 7:**
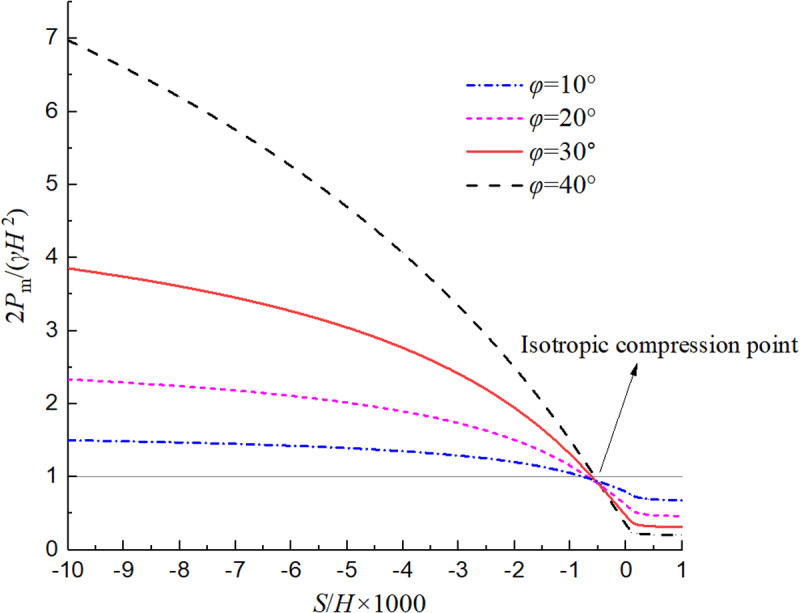
Relationship curve of the normalized *P*_m_ with displacement and internal friction angle.

**Fig 8:**
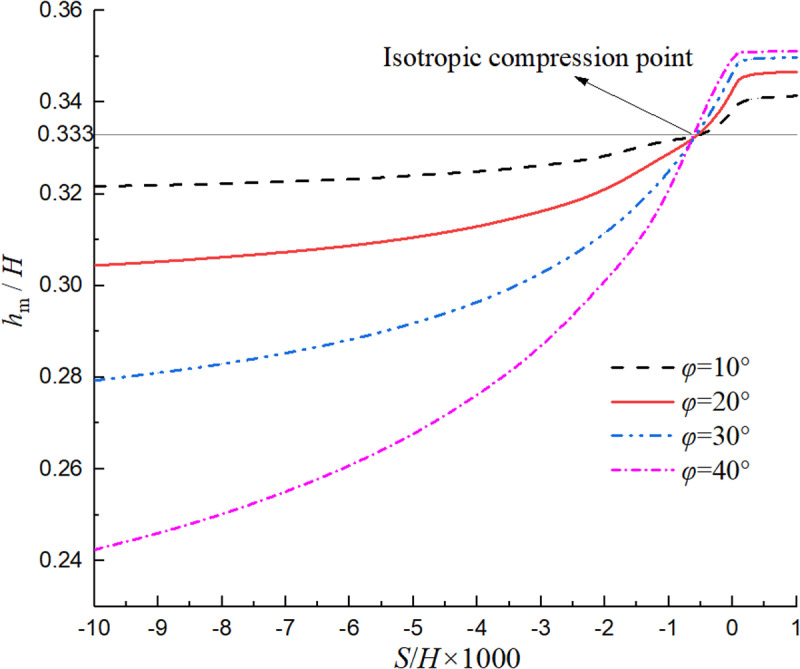
Relationship curve of the normalized *h*_m_ with displacement and internal friction angle.

Fig 7 shows the relationship between the normalized value of *P*_m_ and displacement and internal friction angle. As can be observed, when the soil changes from the passive limit state to the active limit state, the resultant earth pressure will decrease nonlinearly. The variation range of earth pressure with displacement in non-limit passive state is obviously larger than that in non-limit active state, and it will further increase with the increase of internal friction angle. Fig 8 shows the relationship between the normalized value of *h*_m_ and displacement and internal friction angle. It can be seen that when the soil changes from the limit passive state to the limit active state, the position of the resultant force will increase nonlinearly. In the non-limit passive state, the change range of the position of the resultant force action point with displacement is obviously greater than that in the non-limit active state, and the change range will further increase with the increase of the internal friction angle.

It can also be seen from Figs 7 and 8 that the relationships of *P*_m_ and *h*_m_ with displacement and internal friction angle is not linear, mainly because the internal friction angle *φ*_m_ obtained in this paper is nonlinear with the displacement ratio, and the soil arching effect is considered in the process of deriving the earth pressure. The four curves in the two diagrams all intersect at the same point, mainly because *φ*_m_ = 0 at this point, the soil is in an isotropic compression state, and the distribution of earth pressure is consistent with that of still water, that is, 2*P*_m_/(*γH*^2^) =1 and *h*_m_/*H* = 1/3.

In practical engineering, the displacement of the wall generally needs to be limited, which makes it difficult for the backfill behind the wall to reach the limit state. Therefore, the earth pressure theory considering the influence of displacement can ensure that the designed retaining wall is safer and more economical. The unified solution of active and passive earth pressures in non-limit state obtained in this paper can reasonably calculate the earth pressures under different displacements. The calculation formula is concise, the mechanical concept is clear, and the application is relatively simple. The results of this paper can lay a foundation for a deeper understanding of earth pressure, and also provide reference ideas for unifying the theoretical solutions of earth pressure of retaining walls under other displacement modes.

## 6. Conclusions

(1) Based on Mohr’s stress circle, through Duncan-Chang hyperbolic stress-strain model, the relationship between friction angle and displacement ratio is obtained by analogy with the variation law of earth pressure and displacement of retaining wall. By changing the sign displacement ratio, the formula can reflect the friction angle in non-limit active state and non-limit passive state simultaneously.
(2) In the horizontal layer analysis method, the internal friction angle *φ*_m_ and the wall-soil friction angle *δ*_m_ under arbitrary displacement are introduced. Meanwhile, taking the soil arching effect into account, the unified expressions of the active and passive earth pressures in the non-limit state of the retaining wall, the unified expression of the resultant force of the non-limit earth pressure and the unified expression of the height of the resultant force point are obtained. The theoretical solution in this paper is compared with other theoretical solutions and experimental values, and the reliability of the formula is verified.
(3) In the process of changing from the non-limit passive state to the non-limit active state, the resultant force of earth pressure gradually decreases nonlinearly, and the position of the acting point of resultant force gradually increases nonlinearly, both of which are positively correlated with the internal friction angle.
(4) The unified solution of non-limit active and passive earth pressures proposed in this paper can fully consider the influence of displacement. It has a simple formula, a clear mechanical concept and convenient application, which can provide reference for relevant engineers.

## Supporting information

S1 File The values used to build graphs. (ZIP)
